# The Case of Dr. Bartlett against the Erie County Medical Society

**Published:** 1864-06

**Authors:** 


### EDITORIAL DEPARTMENT.

### THE CASE OF DR. BARTLETT AGAINST THE ERIE COUNTY MEDICAL SOCIETY.

(CONCLUDED.)

In a preceding article we have given the facts and the judicial decisions bearing upon the case. At present we propose to make such comments as occur to us, for the purpose of putting the actions, we might say the case, of the society in its true light. We trust it will appear that, if the courts have decided that the law is against it, it certainly has on its side equity; and further, that the facts when interpreted properly will satisfy all reasonable minds that its action could not have been dictated by prejudice, but by nothing else than a high regard for the interests and objects for which it was formed.

The statutes regulating the practice of medicine, the charter of the society granted in 1813, the by-laws of the society, and the code of ethics adopted by it, are all referred to in the progress of the case, on the one hand, by the society to prove its authority, and on the other by the courts as ruling its opinion. Those portions of them therefore, which bear most directly upon it are quoted here.

*Laws of the State of New York relative to the medical profession, Title vii,* § 1: "The president of every county medical society shall give notice, in writing, to every physician and surgeon not already admitted into such society, within the county in which the society of which he is president is situated, requiring such physician or surgeon, within sixty days after the service of such notice, to apply for and receive a certificate of admission, as a member of such society."

The penalty for not applying is forfeiture of license until by special application he is admitted.

*Act of April* 10, 1813, *relating to the establishing of County Medical Societies*, § 14: "That it shall he lawful for the respective societies to make such by-laws and regulations relative to the affairs, concerns and property of said societies, relative to the admission and expulsion of members," etc. "as they or a majority of the members at their annual meeting shall think fit and proper."

It is provided however, that they shall not be repugnant to the by-laws of the State Medical Society, nor conflict with the laws or constitution of the State or the United States.

*The by-laws of the Society, Article V, relating to the admission of members*, § 1: "Every physician and surgeon residing in the county of Erie, of temperate habits, good moral character, and legally authorized to practice physic or surgery in this State, who may hereafter wish to become a member of this society, may be admitted by a vote of *two-thirds* of the members present at a regular meeting."

It may be "inconsistent with the constitution and laws of the State and of the United States" to establish a rule that a majority shall not elect to membership. Perhaps an applicant who failed of a two-thirds vote, but obtained a majority, might have a question with the society as to the legality of his rejection. But it is not material in this case, for the applicant never received a majority vote.

*Code of Ethics adopted by the society, Chapter II, Article I*, § 3: "It is derogatory to the dignity of the profession to resort to public advertisements, or private cards or hand bills, inviting the attention of individuals affected with particular diseases"—"to boast of cures and remedies, to adduce certificates of skill and success, or to perform any other similar acts. These are the ordinary practices of empirics, and are highly reprehensible in a regular physician."

*Code of Ethics, Chapter II, Article IV*, § 1: "No one can be considered a regular practitioner, or fit associate in consultation, whose practice is based on an exclusive dogma."

It is also pronounced "derogatory to professional character"—"to dispense a secret nostrum," for the reason that if good, all members of a beneficent profession should know of it, and if its value lies alone in mystery, such craft is disgraceful, equally, whether it depends upon ignorance or avarice.

From the above quotations it will be seen that in the case of Dr. Bartlett, if as he alleges, no notice was given, the society was at fault, and should guard against a similar omission.

It will be also seen that the society has the power to make by-laws and regulations relative to the admission of members, i. e. it has some *discretion* in regard to the matter, and is not, at least the wording of the act seems to warrant such an inference, obliged to admit to membership any one who seems to have clear qualifications. It has a right to inquire into his character, and it would seem, past and present professional conduct, leaving out of view gross ignorance, immoral conduct, or want of a license. At any rate the law seems to imply authority to exercise some discretion or choice, and as a consequence, some right to exclude. The very right to regulate admissions seems to imply the right to exclude. We make this point, because the language of the court, as we shall see, clearly asserts its right to impose members upon the society; the society having no remedy, but to expel, if any of its by-laws are violated by the members, after their compulsory admission.

But the court says granting the society's right to exclude by a by-law framed under the authority given by statute, no such by-law has ever been framed. If the society has the right, it has not exercised it. It will be seen above, that the only by-law relative to admission of members, declares that any physician or surgeon in Erie county, of "temperate habits, good moral character, and legally authorized to practice physic and surgery in the State," may become a member, if he obtains the requisite vote.

There is nothing here which can be applied to the case of one who either is, or has been engaged in irregular practice. It is not specified as one of the conditions of admission that the applicant shall not be at the time, and shall not have been guilty of professional misconduct. If it will give the society authority to exclude from future membership, one who has been guilty of professional misconduct, a by-law to that effect should be framed at once.

It will be moreover apparent to all who read the advertisement included in the preceding article on this subject, that by the code of ethics the publication of such is derogatory to the dignity of the profession and highly reprehensible. It is an offence against the regulations which govern the conduct of all high minded and honorable physicians, and is an imitation of empirics. No man of ordinary intelligence could allege ignorance of its nature, or expect indulgence on the score of its being an indiscretion. The sentiments and the written rules of the profession are very widely known, and any man having acquaintance with medical men, or students, could not have failed to have been made aware of them.

The defence of the society in the second trial at the General Term of the Supreme Court has been briefly alluded to. It is due to the legal gentleman who very ably defended the case, to state more fully the grounds of his defence. It will be remembered that in the first trial at the Special Term, the case was argued as if the application had been made to the court in the first instance, and the question of admission was to be determined by it. The defence insisted that the question before the court was not whether the court should order the admission of the applicant, but whether the decision of the society should be reversed. That is, the society had passed judicially upon his claims and rejected him; 'twas for the court to sustain or overrule the decision. This would give to the society a sort of judicial capacity, and would bring into consideration the rights of the society as a corporate body. The society as a corporate body has powers granted it by statute; it can have a seal, can hold property, grant licenses to practice, examine students, and make rules for the admission and expulsion of members. It acts as a quasi-judicial body in the exercise of an ample discretion, vested in it by the Act under which it was incorporated. If the court interfered at all with it, it could only do so as it would in the case of any other judicial body, upon a question addressed to its judicial discretion. This point was argued upon the general provisions of the statute relating to corporate bodies. The common law gives corporate bodies no right to expel members; it is only derived from express authority granted by the charter. In a case of expulsion a court would not grant a mandamus unless a clear case of abuse of power was made out. The right of expulsion is a right given by statute, and when mentioned in the charter courts have heretofore in most cases not interfered by mandamus. But in the case of the society the right of expulsion is mentioned also with that of admission, the society having authority over one as well as the other. It seems therefore that the intent of the statute was to vest control of both admission and expulsion in the society, else it would not have been expressly mentioned. Not absolute control, but such as would amount to an ample discretion. In all cases some abuse of this discretion should be shown, as for instance, a denial of a strict legal right of admission, to justify the courts in interfering. It does not seem that the court considered at all the question of abuse of discretion, on the part of the society. It assumed the right of compelling admission, and if it saw fit to deny the writ of mandamus, it would be on the grounds of expediency, not of right.

The defence also maintained that the writ of mandamus was not the proper process, it being applied for, where the party has a right to have anything done, and has no other legal means of compelling its performance. It will not do for cases of doubtful right. It is a legal remedy, not an appeal to the equity of the courts. No party is entitled to it, unless he has a clear legal right to demand what he asks. It cannot be claimed that a practitioner has under all circumstances a clear legal right of admission, especially in the case under consideration. If that were the case it would defeat the evident intent of the statute, granting the society control over admission and expulsion of members. Moreover when a court, or officer, or an inferior tribunal has acted judicially, or has exercised a discretion, the court cannot correct that discretion by mandamus.

To the foregoing attempt to state the points of defence may be appended the following quotation which will present the matter more forcibly: "'The writ will not be allowed in cases where corporations, and ministerial and other officers have acted judicially, nor where they have a discretion in regard to the performance of an act, and have exercised the discretion conferred upon them. But if they refuse to act when required by law the court will compel them by mandamus, and the operation of the writ is the same in these respects as when directed to subordinate judicial tribunals.' These cases (cases cited, but reference omitted here) hold that corporations may act judicially. They do so in this case. These corporations (county medical societies) are made the tribunal to judge as to the admission of members by the statute. The authority is wisely lodged there. They are presumed by the statute to be composed of men of learning, wisdom, and integrity. The preamble to the Act of 1813, asserts that the object of these societies is the diffusion of true science, and particularly the knowledge of the healing art. What provisions or action as to membership will most promote this object must, as a general rule, be much better known to them than to any court; and when this tribunal has once passed upon an application for membership, this court should be very slow to reverse its decision, and never unless a clear case is made out, of an abuse of the discretion conferred upon the original tribunal. Had the society refused to take any action on his application, it could, no doubt, have been compelled by mandamus to do so. But having taken action and judicially decided the relator's case, the court if it can interfere at all, can only do so in case of an abuse of discretion, i. e. practically not at all. We concede that the by-laws and all other acts must be reasonable, that they must not be vexatious, unequal, oppressive, or manifestly detrimental to the interests of the corporation; but we contend that, in the first instance, the corporation is, in cases like that before the court, the judge of what is reasonable, and that that judgment cannot be disturbed unless in case of manifest abuse."

This very imperfect statement of the main points in the defence, will doubtless be of interest to the members of the society, few of whom were present at the trial. They did not weigh with the court, as has been apparent in what has gone before. The question of right to compel admission was assumed by the court, as will be seen by reference to the decision of Judge Daniels, unless forfeited in some way. But the court held that it was not forfeited. For though he had been guilty of practices condemned by the by-laws of the society, and in violation of the code of ethics, his right of admission was not thereby affected, because those by-laws and regulations were only for the government of members; he not being a member, they did not apply to him. They could not operate to exclude him because, though a violation of them would subject a member to discipline or expulsion, they had not expressly stated that acts, which would be a violation of them in a member, should when committed by one not a member, exclude him from future membership. The relator therefore, though guilty of irregularities, and of acts which would have been violations of by-laws had he been a member, having abandoned all such practices, and declared his intention to conform to the general regulations governing the conduct of regular practitioners, and not only declared his intention to conform, but actually conformed, (no proof to the contrary being presented,) was entitled to membership. Whatever control the society might have over the subject of admission, it had not made any by-law which would apply to his case.

But it seems from the case cited in the opinion of the court, that the right to admission did not depend upon the abandonment of irregular practice. The case cited, that of *ex parte* Paine, was one where the applicant was at the time of making application for membership, in the continuance of unprofessional conduct, and declared his intention to adhere to it. The court denied the writ of mandamus, not because the right of admission did not exist, or had been forfeited, but because the writ could be made ineffectual by an immediate expulsion from the society. That is, it denied the writ, not because no legal right existed, but because it would have been unwise or impolitic. If the society has no better case than this, there is very little advantage in statutes.

It is true it can expel a *member* guilty of offences against its by-laws; but it cannot prevent an irregular practitioner, after years of empiricism, from participating in the benefits of membership in the society, and from being ranked with regular and honorable physicians; provided only he make a hasty repentance, and declare that he means to be guilty of no more misconduct. The court claims that it can force him upon the society, and it can overrule any attempt to exclude. With such a man, high-minded physicians must associate at the bed-side, or be guilty of a breach of the by-laws. Carrying out the decision of the court to its possible limits, would make the profession a respectable refuge for damaged reputations, and disappointed empirics. It may be strictly legal, but it is far from being equitable. The plainest common sense would have no difficulty in deciding that there was very little justice, if much legality in it. The society must submit to the decision of the tribunal before which the case will soon come. If it be adverse, it has no remedy. But it will not increase its respect for that mighty and weighty thing in law, viz: a precedent, which holds such sway over judicial minds. Common sense men make laws, in the main, for the ends of justice, but the courts construe them. It is possible that the intent of the law may be violated by construction. The society is impressed with the idea that the intent of the statute giving it power to regulate admissions is plain. Whether it will be held so or not, is to be determined. The judges at the General Term held that the society could not act judicially in this case, because the relator not being a member, it had no jurisdiction over him to try or condemn him.

The last paragraph of the opinion of the court should not be passed over without comment. It states that no opportunity was given the relator to explain, etc. This is not true, as he had an interview with the committee. But allowing it to be true: if the society had no right to sit in judgment on him, why should it give him a hearing? Moreover the court held that it could not do so because the by-laws declare that "no candidate shall be present until the question of his admission be determined by the society." All which in plain language is thus. The society was at fault for not giving a hearing: but it could not because its by-laws forbade. The society was wrong for not doing what it had no right to do. The suggestion, that "if the society had been desirous of dealing with him as an offender against its laws," "he should have been received as a member upon his own application, and then it could have enforced the by-laws against him, if he afterwards professionally misconducted himself;" may strike the society as a very ingenious way of dealing with any similar case. It seems something like this, if you wish the door kept shut, open it; if penalties for misconduct are to be enforced, they must be remitted. If the society desires to keep a man from membership on account of misconduct, admit him, and then trust to being able to find cause to turn him out. The society must lie low as it were; give no hint, by making inquiries as to the past, of its future purpose; encourage in other words misconduct; then when the unsuspecting applicant is fairly in, punish him by expulsion, if his offence will warrant. But, on the other hand, such action on the society's part would make the way quite easy and pleasant for those who wish to make an experiment of empiricism. They could try it, and if it disappointed, or if they tired of it, they could reform and be sure of membership, because the society would desire to get them under control.

In the opinion given by Judge Davis there is a little confusion as to which application is meant, that to the court, or that to the society. He says, "had that publication been continued at the time of his application, I should regard the action of the society as entirely justifiable." The connection would lead to the inference that the application to the society was meant. If so, the Judge was not correct as to his facts. It will be seen that the first advertisement was kept in the paper till a few months before his application to the society, and the second, which is equally objectionable in view of the one which preceded, up to the time of the application, June, 1859.

We have thus, at the risk of wearying our readers, considered the legal aspect of this case thus far. From it the society may be led to this most consoling conclusion; that it is likely to have an irregular practitioner forced upon it, despite any right or power it has in the premises. Let a practitioner prove his license, and go before the court, and it will assumed the right to compel his admission to the socieiy. If he is practicing irregularly at the time, the court may think it unwise to grant a mandamus. If he has abandoned irregular practice, though he may have sinned long and thoroughly, it will most probably grant the writ, for it will be lenient to such "indiscretion." A licensed physician has an absolute right of admission, no matter what professional irregularities he may have been guilty of, and the society has no discretion in the matter of admitting to membership. This is the present aspect of the case.

We must beg indulgence for a brief consideration of the facts in this case, and the inferences which seem fairly deducible from them. The society disclaims all personal bias. This is a question of example, of professional policy, so to speak, in which the public is as much interested as the profession. It concerns the usefulness as well as the respectability of the profession. As a legal question, novel in some of its features, the society is interested to have a decision beyond appeal. It is moreover not indifferent to the equities of the matter. It is truly desirous of acting justly; and equally desirous that its rights should be determined and respected.

The facts of the case stated briefly are as follows: Dr. Bartlett, a recently graduated physician comes to Buffalo, and without making application to to the society for membership early, begins his practice by a public advertisement, announcing himself a specialist, and a follower of the method of a well known quack. This advertisement he continues nearly four years, and then substitutes for it another, less objectionable, but still in public journals. Four years and over, after his coming to the city he applies to the society for membership, stating that he had abandoned special practice and public advertisements, and had engaged in, and meant to continue to devote himself to, general practice; not claiming any especial skill, or using secret remedies, or associating with irregular physicians, In other words, conforming in all respects to the rules and ethics of the profession. One year after his application was made, it was rejected by the society, and more than two years after, he applied to the court for a process to secure his admission. His admission was twice ordered by the court, and twice the society has continued the case by appeal. The matter now rests with the Court of Appeals, and the decision will be final when given.

Now we suppose that a favorable interpretation of the facts would be, that a young medical man, a stranger, anxious to be engaged in business, took what he deemed the best means to advance his own interests, without thinking to bring down upon himself the censures of the profession; in mere thoughtlessness as it were, or ignorance perhaps. As soon however as he found he was conducting himself in an unprofessional manner, he hastened to discontinue all acts which could be so construed, and sought in good faith to associate himself with the profession in a regular way, desiring to comply with, and be governed by its rules and ethics. Accordingly he made his application, explained his conduct, announced his intentions, and desired, perhaps expected to be received. But the society being slow to receive him he began to feel aggrieved, and upon a final rejection, resolved to get justice through the law. Now such an interpretation we say might, by those favorably disposed, be put upon the case, and the applicant be put in the light of one whose rights had been denied; and the society in the position of being unwilling to grant a simple act of justice.

But another interpretation of the facts more favorable to the society might be made, which would entirely free it from any imputation of injustice or even vindictiveness, and place it in its proper position as a jealous guardian of the welfare and honor of the profession.

Accordingly the facts would bear this interpretation. A young man, just admitted into the profession, on coming to the city in which he intends to reside, shows himself utterly indifferent to those things which are usually the first care of those entering professional practice, viz: to join the regular medical organizations, and to observe scrupulously rules and ethics. Young medical men are usually more scrupulous in these matters than older ones; and have generally high ideas of professional etiquette and honor. They are usually anxious to associate with, and secure the approbation of their elders. Of the many recent graduates who have located themselves in this city, meaning to practice regularly, all have early sought admission to the society. The fact that Dr. Bartlett did not, gives rise to a presumption, that, at first he intended to ignore the regular profession; not mere thoughtlessness. Secondly, he advertises in the public journals, an act of itself such a violation of all the rules and customs of the profession as no young man just entering it with high ideas of its honor would for a moment entertain. But further, the method he proposes to use and the special diseases he intends to treat, both create presumptions against him.—What young man proud, as young men are usually, of being admitted to an honorable calling, would for a moment propose as his model a well known quack? What young man of modesty, and above all inexperienced, would set up a claim to superior skill in certain diseases, when from his very circumstances he could not possess it? We cannot believe that such acts could consist with a high regard for the profession, or with an intention to observe its regulations. In a young man, an advertisement to the public, announcing his intention to treat special diseases is downright presumption; because skill is only acquired by experience, it does not come by nature. No doubt a young man who had made a single disease a separate study would be better prepared to treat it, than a young man of equal abilities who had studied all diseases. But he would be far inferior to most practitioners of experience, who would know much more practically of the single disease to which he proposed to give his attention, although they had not made it a specialty. The public does not see it in this light, and therefore if a man advertises as a specialist, it is inclined to give him credit for superior skill, though a little reflection would convince it of its error. For this reason the ethics of the profession condemn as dishonorable advertising specialties, as being attempts to get business greater than is merited by knowledge or experience.

But it is leniently construed to have been done from no motive of this nature, but thoughtlessly or ignorantly, and discontinued as soon as it was made to appear that it was unprofessional. The applicant himself, and those charitably disposed towards him, must not be surprised if the society is inclined to put quite another construction upon it. It seems not to be an unfair presumption that the advertisement was continued as long as was needed to answer its purposes. It brought Dr. Bartlett before the public as a specialist, it created as all such things do, most undeservedly usually, a belief in his superior skill in the diseases he proposed to give special attention to, and of course brought more or less practice. Having been continued awhile, it spread widely enough for the purpose of his supposed excellence, and could be without detriment withdrawn. He will not undertake to deny that he is still employed as a specialist in consequence of his advertisement. The society assumes that it was withdrawn because it had answered its purpose. Certainly, if a sense of the impropriety of the act was the cause of its being withdrawn, four years seems rather a liberal allowance of time for the nature of the act to appear. Now it may be that an additional motive was a desire to still keep within the possibility of future recognition by the profession. It probably began to be apparent that this was of considerable importance. For no doubt he often found himself in an unpleasant situation, being cut off from consultation with members of the society. If the friends of patients, especially after he undertook general practice, asked him to seek advice, whom should he call upon? No member of the society could consult with him, His isolated position was unpleasant and detrimental, much more so perhaps than he was prepared for when he, as the society assumes, early neglected professional regulations.

The profession is often accused of being harsh in its judgments, and unreasonable in its opposition to anything which looks like quackery. It is not an unfair spirit, when the peculiarities of medical practice are considered. When it is considered by what easy methods a reputation may be obtained, altogether disproportionate to any skill or knowledge, from the fact that the public is inclined to believe in unfounded pretensions, simply because they are put forward. Nothing can be more creditable than the nice sense of honor which a true professional man feels, in circumstances where by acts which the body of the profession have justly stamped as unbecoming, and even dishonorable, so much personal advantage can be gained, at least for a time, and a reputation for superiority over associates established, when in fact none exists. The profession is jealous of asserted superiority, justly so, because it has an undue influence. Now a public advertisement is a claim to superior skill, and reaps the fruits which belong to it.

No single member of the society desires to act unjustly towards Dr. Bartlett, or indulge harsh judgments towards him. Some members even, and four of a committee of five, were prepared to put a very favorable construction upon his conduct and accept him as a member. But human actions are usually prompted by mixed motives, and in judging of them it is usual to ascribe them to those which seem most likely to have had the greatest influence. It is not always possible to judge correctly of motives from actions, but it is the custom of men. The society has done no more. It had not determined that the exclusion should be final. Time and good conduct might, and probably would have removed all objections to admission. But we are all incredulous of reform from sincere regret of having erred, when, at the same time, there are strong urgings of personal advantage to help it on. We are apt to feel that the latter is the more weighty, especially if facts make it plausible in theory. The profession and the public often take diverse views of these matters. To the latter, a public advertisement seems no unpardonable offence. The profession condemns it, mainly because it is the method of quacks, and very often the less deserving the person, the more boastful the advertisement. The public cannot so readily see that as with quacks, those who take this method have generally poorer abilities for the thing they promise to do, than many others. It must be understood that this relates only to the practice of medicine under present professional regulations.

It may be well here to say a few words regarding specialists. There is with the great body of the profession a feeling of decided opposition to them. The codes of ethics condemn them. They find no favor in public assemblies of medical men. Now why is this? In the first place all, or almost all quacks are specialists. But it is no proof that a thing is in itself bad, because unworthy men practice it. In the second place, it often is a resort of incompetent and inexperienced medical men to gain business which they could not otherwise get. There may be other reasons, but these are the most obvious. Now it may be well to consider whether, because quacks and ignorant or inexperienced medical men resort to special practice it should be so condemned by the profession. There can be no objection to the practice of a specialty when from study and experience a man is well qualified to treat particular diseases. Skill in the treatment of special diseases, when it really exists, ought to bring to the possessor abundant employment. No one can object to the preference given to a really skillful physician in any branch of practice. The reputation and success are legitimate; the result of care, investigation, study and experience. When put in this form there would probably be less objection on the part of the profession, for it has always honored excellence. But it is because special practice offers such opportunities for utterly worthless and incompetent men to gain a great reputation with the public, to abuse its credulity, and do violence to its purse and persons, that the condemnation has heretofore been so decided. The matter has so gone, that the mere announcing of a specialty creates at once a presumption of quackery, ignorance, or a dishonest desire to thrive. Young men, the ink on whose diplomas is hardly dry, announce an intention of giving particular attention to and of course a desire to practice in special diseases. It is perfectly apparent that practically their treatment is worth but little, that is, no more than that of many young but inexperienced men of good abilities. But with the public it may outweigh in the treatment of a special disease the authority of grey hairs, and large general experience in the special as well as other diseases. The profession knows that these claims to special skill may pass for more than they are worth. That if it remains silent or approves, it gives value to attainments in many cases, not in all, not at all above those of the general practitioner; often indeed inferior. Therefore it sets its face against specialists,

But it is worth while to reflect, whether if it gave them its approval there would not be a gain. Whether it would not diminish the success of quacks and keep pretenders within bounds. For after all, it is a belief in the possession of superior skill that leads sensible men and women to trust themselves in their hands. Let the public once fully understand that thoroughly educated and competent specialists are to be found in the profession, and they will not run to quacks for that benefit which they believe the experience or studies of the regular profession will not give. Of course it would not be reasonable to expect the profession to endorse special practice before special merit existed. It must be acquired, but when acquired let it be recognized and encouraged. In fact, real merit could not be deprived of its legitimate success. But, by allowing real ability to work in special cases, bogus merit will be much less likely to pass for genuine, and almost monopolize specialties. The remedy is somewhat with the profession itself. It seems proper that specialists should be required to give exclusive attention to their particular branches, and resign general practice. Indeed that is but a just concession to professional sanction. Perhaps this can only be done in the larger cities, but the larger cities are also the productive fields which quacks so profitably cultivate. In cities where specialists are not under the ban of the profession at large, quacks do not meet with the greatest success. This is not meant to imply a sanction of public advertisements, or a boastful claim of superior skill as social opportunities favor. It is quite as objectionable to proclaim from house to house and to ears professional and unprofessional, success and cures, or even one's specialty, as to put it into the newspapers with certificates of Doctors of Divinity, mayhap. Special practice demands a man of truth, and honorable motives, above playing upon fears or exaggerating ailments, above the dishonest desire to succeed beyond his merits; more perhaps than general practice.

In this matter, touching the best method of dealing with the subject, there are acknowledged difficulties. But it appears reasonable to suppose that a less stringent condemnation of specialists, would result to the injury of quackery, and the benefit of the profession. It would not be difficult to show how badly many diseases which are selected for special practice, are treated by the general practitioner—to instance a single class, viz: diseases of the eye—and how much better they fare with competent specialists.—That being the case specialists will practice, and it is much better that they should be restrained by professional rules, than allowed to run wild, vexing and being vexed. In Europe special practice is sanctioned by the profession. We should remember that to specialists we are indebted for many of the valuable monographs which of late years have been written; and that it is special practice which makes such excellence in writing on special subjects possible.

In conclusion we may be allowed to state that in the case which has been considered at such length, the objections to the reception of Dr. Bartlett rested upon acts which even the court calls "gross empiricism." But the adoption of a specialty is certainly not to be considered so objectionable as advertising it. Nor is the treatment of a special disease so much to be condemned, as professing to follow the method of a quack. The profession may soon be brought to countenance a specialist, if there is a belief in his special merit. But the other acts are, in their nature, unbecoming and reprehensible, and cannot be made less so by any attempt at explanation or palliation.

